# Digital Strategies to Accelerate Help-Seeking in Youth With Psychiatric Concerns in New York State

**DOI:** 10.3389/fpsyt.2022.889602

**Published:** 2022-05-16

**Authors:** Michael L. Birnbaum, Chantel Garrett, Amit Baumel, Nicole T. Germano, Cynthia Lee, Danny Sosa, Hong Ngo, Kira H. Fox, Lisa Dixon, John M. Kane

**Affiliations:** ^1^The Zucker Hillside Hospital, Northwell Health, Glen Oaks, NY, United States; ^2^The Feinstein Institutes for Medical Research, Manhasset, NY, United States; ^3^The Donald and Barbara Zucker School of Medicine at Hofstra/Northwell, Hempstead, NY, United States; ^4^Department of Health Systems and Population Health, University of Washington, Seattle, WA, United States; ^5^Department of Community Mental Health, University of Haifa, Haifa, Israel; ^6^Department of Psychiatry, New York State Psychiatric Institute, New York, NY, United States; ^7^Department of Psychology, Barnard College, Columbia University, New York, NY, United States

**Keywords:** digital advertisements, early intervention, youth mental health, help-seeking, social media, pathways to care

## Abstract

**Background:**

Mental illness in transition age youth is common and treatment initiation is often delayed. Youth overwhelmingly report utilizing the Internet to gather information while psychiatric symptoms emerge, however, most are not yet ready to receive a referral to care, forestalling the established benefit of early intervention.

**Methods:**

A digital outreach campaign and interactive online care navigation platform was developed and deployed in New York State on October 22, 2020. The campaign offers live connection to a peer or counselor, a self-assessment mental health quiz, and educational material all designed to promote help-seeking in youth and their allies.

**Results:**

Between October 22, 2020 and July 31, 2021, the campaign resulted in 581,981 ad impressions, 16,665 (2.9%) clicks, and 13,717 (2.4%) unique website visitors. A third (4,562, 33.2%) completed the quiz and 793 (0.1%) left contact information. Of those, 173 (21.8%) completed a virtual assessment and 155 (19.5%) resulted in a referral to care. The median age of those referred was 21 years (IQR = 11) and 40% were considered to be from low-income areas. Among quiz completers, youth endorsing symptoms of depression or anxiety were more likely to leave contact information (OR = 2.18, 95% CI [1.39, 3.41] and OR = 1.69, 95% CI [1.31, 2.19], respectively) compared to those not reporting symptoms of depression or anxiety. Youth endorsing symptoms of psychosis were less likely to report a desire to receive a referral to care (OR = 0.58, 95% CI [0.43, 0.80]) compared to those who did not endorse symptoms of psychosis.

**Conclusion:**

Self-reported symptomatology impact trajectories to care, even at the earliest stages of help-seeking, while youth and their allies are searching for information online. An online care navigation team could serve as an important resource for individuals with emerging behavioral health concerns and help to guide the transition between online information seeking at baseline to care.

## Introduction

Mental health concerns in transition age youth are common and approximately 20% have a diagnosable mental illness (1–3). Symptom onset frequently occurs during formative years of adolescent and young adult development, and interferes with the establishment of healthy educational, vocational, and social foundations. While early intervention improves the likelihood recovery (4, 5), treatment initiation is often delayed (6), resulting in worse outcomes with long-lasting deleterious consequences persisting well into adulthood (7, 8).

Treatment initiation delays are multifaceted and include (i) demographic characteristics (9, 10) including age, sex, race, income, and health insurance status; (ii) systemic factors (11, 12) such as ill-defined pathways to care (iii) illness-related factors (13) such as speed of symptom onset; and (iv) environmental factors (14, 15) such as perceived stigma and level of mental health awareness within the family and the community. There is therefore no single existing strategy that would completely address this public health challenge, as it involves a constellation of factors unique to each individual. Novel, personalized, and nimble efforts are necessary to accomplish this goal.

Technological innovation, harnessing the established power of digital media, offers the prospect of facilitating treatment initiation by proactively identifying and engaging youth with behavioral health concerns online. Youth with mental health concerns overwhelmingly report utilizing the Internet first and most frequently to gather information while psychiatric symptoms emerge (16–18). Further, searching online represents one of the first proactive steps toward treatment initiation (19–21). However, the majority of youth searching for mental health related information online, including those who may be at risk for psychiatric disorders, report that they are not yet interested in receiving professional care (22), forestalling the established benefit of early intervention.

Advertisers routinely and effectively use the Internet to micro-target specific consumer segments directly beyond the capabilities of traditional media (23), however, limited efforts have focused on applying available technologies to engage help-seeking youth (and their allies) online and refer them to appropriate resources (24). Toward this goal, our team has developed a comprehensive care navigation platform (NYWell) designed exclusively to promote early intervention by identifying and engaging youth online with behavioral health concerns and to expeditiously link them to local resources. Leveraging search engine and social media-based advertisements, the project encourages participants to interact with our care navigation team online by offering peer-led support and guidance, as well as a virtual assessment conducted by a live clinician, and referral to care, if indicated.

While the project’s primary research objective is to examine its effectiveness at reducing the duration of untreated psychosis for youth with first episode psychosis (FEP), we have, in the process, interacted with thousands of youths and their allies with a wide variety of behavioral health questions and concerns. The trial is currently active and the goal of this paper is to report on the pathways to care for all NYWell visitors from ad impression to receiving a referral to care based on the first 9 months of the project starting from the date of deployment (October 22, 2020) to July 31, 2021. We hypothesize that rates of engagement, assessment, and referral for both youth and their allies would vary based on self-reported psychiatric symptoms.

## Materials and Methods

**FIGURE 1:**
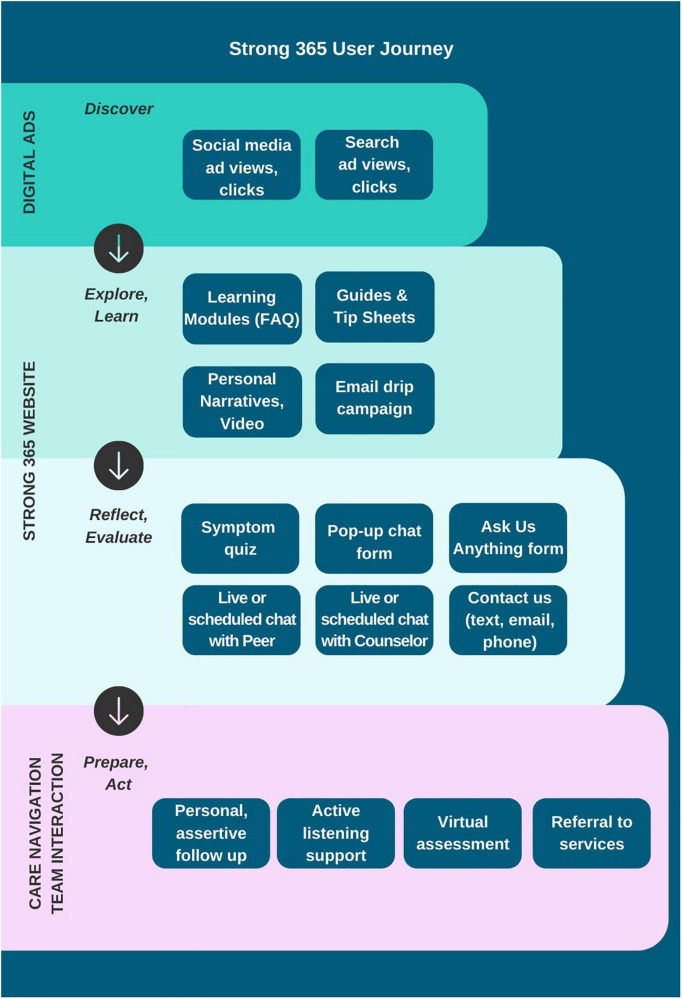
User experience from ad to referral.

Northwell Health’s Early Treatment Program (ETP) and Strong 365, a non-profit dedicated to raising awareness about early psychosis intervention, collaborated on the development of a digital outreach campaign and interactive web-based platform aimed at understanding the role that digital media can play in promoting help-seeking for youth in the early stages of psychosis (Figure 1). This study was funded by NIMH (R34MH120790) and was approved by the Northwell Health IRB (#19-0266). Following a stepped wedge randomized controlled design, the campaign began running in select regions of New York State (NYS) on October 22, 2020, and expanded to the entire State of New York on October 25, 2021. The complete trial is due to run for a total of 18 months ending on April 22, 2022.

NYWell’s content and design were created using participatory design principles (25). The project’s multidisciplinary team include an advisory panel with lived experience, psychiatric researchers, peer support specialists, clinicians, social marketers, web developers, user-experience designers, graphic artists, and digital advertisers. Though our target audience is youth with early psychosis and their allies (parents, educators, primary care physicians, mental health care professionals, community/faith leaders, and friends), a key insight gained in preparatory interviews with stakeholders at the outset of prototyping was that the terms used to describe their early experiences with psychosis were broad (for example, stress, sleep, feeling different). Further, changes in mood and anxiety are often noted to be among the first symptoms to develop in emerging psychotic disorders (26). We thus strategically created an online environment that would be relevant and engaging to individuals with any question or concern pertaining to mental health. Youth and their allies also reported a desire to find information “all in one place,” with simple language, easy navigation, and a clear set of potential actions, from a trusted source. Based on these insights, we aimed to construct an inviting online experience, for those who may themselves not yet be fully aware of, or ready to disclose (due to suspiciousness or paranoia, for example), psychiatric symptoms, as well as those who preferred to remain anonymous.

### Campaign Overview

We developed and simultaneously deployed two separate digital media campaigns with advertising and web content targeting two audience segments. The first campaign targets youth searching online for mental health related information on behalf of themselves (Youth group). The second targets concerned caregivers and allies (Ally group). In order to provide the best opportunity to reach the intended audience, digital advertisements were placed on Google, Facebook, and Instagram platforms. Ads are demographically, geographically, and interest-group targeted, and appear in response to online activity conducted by each user. Users click from an ad to the campaign website, where they can engage with the NYWell care navigation team via text, live/synchronous chat, asynchronous chat, email, or phone. NYWell is accessible both via desktop and mobile.

#### Digital Ads

**FIGURE 2:**
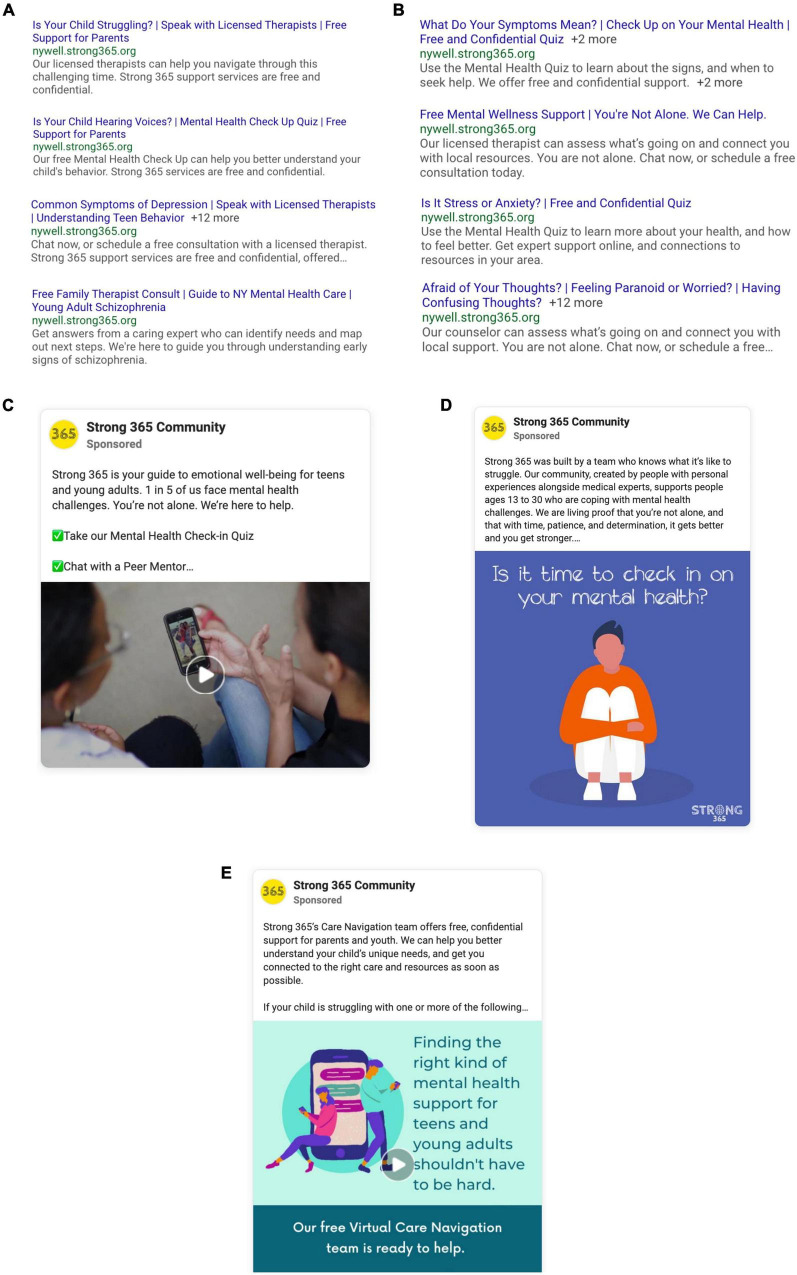
Sample social media and search engine ads. **(A)** Example Search ads targeting allies such as parents, educators, medical professionals who are searching for mental health-related information. **(B)** Example Search ads targeting youth who are searching for mental health-related information. **(C)** Example social media video ad featuring a young person with lived experience sharing their story. **(D)** Example social media graphic ad inviting young people to take the quiz. **(E)** Example social media animation ad targeted toward parents of teens and young adults.

More than 4,900 keywords (purchased search terms) and 240 search engine ads were created and tested, spanning numerous symptom/experience and health-behavioral categories (Table 1). Each ad consists of a headline, description, and call to action. Keywords and ad content were drawn from user interviews, analysis of search trends in NYS, and the performance of prior pilot campaigns (24). Additionally, 75 image and video-based ads were placed on Facebook and Instagram (Figure 2). Based on platform user data, a younger demographic was primarily targeted on Instagram, and an older demographic on Facebook (27). Beyond demographic and geographic targeting, the following interest groups were included: educators, parenting, medical and mental health professionals, LGBTQ + , gender queer, sports and coaching, college, high school, child protective services/foster care, juvenile justice, and homeless youth.

**TABLE 1:** Top performing search engine ad content (by clicks).

	Top 10 Ad groups	Target	Clicks	Top performing keywords	Top performing headlines
	(By clicks)	audience	N (%)	(By clicks)	(By clicks)
1	Information about mental health conditions, promoting quiz as a next action	Youth	3,387 (43%)	Psychological tests Extreme anxiety Anxiety quiz Mental illness test Depression symptoms	What Do Your Symptoms Mean? Check Up on Your Mental Health Free and Confidential Quiz
2	Ally focused ads promoting quiz as a next action	Ally	1,775 (23%)	Mental health assessment Symptoms of depression Anxiety test Schizophrenia disorder Mental illness test	What Are Depression Symptoms? Understand Anxiety Symptoms Early Signs of Schizophrenia
3	Depression-focused, promoting quiz as a next action	Youth	859 (11%)	Mental depression Depression test Clinical depression Depression Help for depression	What is Major Depression? Do I Have Clinical Depression? Free and Confidential Quiz
4	Parent-focused ads promoting quiz as next action	Ally	702 (9%)	Mental illness Symptoms of depression Anxiety symptoms Depressed child Child sleep problems	Is Your Child Struggling? When To Seek Help For A Child Free Support for Parents
5	Speak with a therapist	Ally	327 (4%)	Symptoms of depression Free mental health quiz Mental health evaluation Child not sleeping Depression test	Is Your Child Struggling? Speak with Licensed Therapists Free Support for Parents
6	Free guidance and consultation	Ally	245 (3%)	NYC mental health hotline New York mental health resources Mental health symptom quiz Mental health assessment Young person mental health	Speak with Licensed Therapists Free Support for Parents Free Consult for NY Parents
7	Mental health-related experiences as described by youth	Youth	190 (2%)	Am I depressed test Am I depressed quiz Bipolar quiz Compulsive thoughts Obsessive thoughts	What Do Your Symptoms Mean? Check Up on Your Mental Health Free and Confidential Quiz
8	Information about mental health conditions, promoting peer consultation as a next action	Youth	164 (2%)	I have schizophrenia Mood disorder test Bipolar quiz How do you know if you have bipolar Psychiatry help	What Do Your Symptoms Mean? Check Up on Your Mental Health Talk to Certified Peer Mentors
9	Information about mental health conditions, promoting therapist consultation as a next action	Youth	107 (1%)	Disorder test Psychological tests Coping with stress Mental check Free therapy	Mental Health Support Online Free Mental Health Assessment Our Therapists Can Help
10	Schizophrenia-related inquiries	Ally	42 (1%)	Child schizophrenia Teenage schizophrenia teenage schizophrenia stories Symptoms of schizophrenia in teens Child schizophrenia test	Teen & Young Adult Specialists Early Signs of Schizophrenia Symptoms of Schizophrenia
Total clicks in top 10 ad groups			7,798		

#### Campaign Website

For each campaign (Youth, Ally), an audience specific website was developed (see Supplementary Figure 1). To optimize visibility, the websites were designed to focus on core messages derived from prior pilot initiatives and found to be most relevant to information seeking individuals (24). These include accurately identifying and understanding early signs and symptoms of emerging psychiatric illness and finding local support (28). Based on feedback from lived-experience co-designers, the content is displayed in a simple yet informative, and engaging manner utilizing text, images, videos, and empowering personal narratives. The site is translatable into any language in recognition of the language diversity among the population we aim to serve. The site sought to encourage anyone with psychiatric questions or concerns to engage with the care navigation team in a format that met their desire for anonymity, confidentiality, immediacy, and ease of scheduling a conversation at a convenient time. The site facilitated connection with a compassionate peer listener who could help navigate the mental health system, or a licensed mental health counselor who could offer context and suggest a set of personalized next steps.

Once on the website, psychoeducation is offered as a tool to enhance appreciation for the early warning signs and symptoms of mental illness as well as the benefits of early intervention. Educational materials include Frequently Asked Questions (FAQ), blog articles, tip sheets, videos, and short written narratives of young people sharing their personal experiences. Users are also offered the option to sign up for an email newsletter written by team members with personal lived experience, which is set up to regularly deliver wellness tips, personal stories, and educational content over the course of several months. We offer multiple paths to take action based on user preferences, including a chatbot that pops up on the site for first-time visitors asking “How we can help?,” inviting them to leave their contact information for same or next-day follow-up, and an “Ask us anything” form, in which a user enters a question that is answered by the care navigation team same or next-day.

#### Mental Health Check-In Quiz

A mental health quiz offers the opportunity for self-evaluation. We developed and deployed a brief quiz that was designed to serve as an engagement tool, rather than a diagnostic screener, with limited clinical utility. We encouraged all quiz takers to interact with the care navigation team upon completion to learn more about their own mental health. We thus selected a broad-based approach asking about symptoms of anxiety, depression, and psychosis. The quiz contains a total of 21 items including 4 questions adapted from the PRIME screen (29), which screens for psychosis, 2 questions adapted from the Patient Health Questionnaire (PHQ-9), which screens for depression (30), and 1 question adapted from the Generalized Anxiety Disorder Questionnaire (GAD-7), which screens for anxiety (31). The Ally campaign also includes a 21-item quiz consisting of parallel questions that were adapted to be relevant to caregivers’ perspectives of emerging symptoms in a loved one (“your loved one” as opposed to “I”). We additionally included optional questions regarding demographics, motivations for seeking information or support, their interest in a variety of possible next steps as a means of assessing readiness to take action, as well as how well they were able to engage in the things that are most important to them such as work, school, and relationships, on a scale from 1 to 10, with 1 representing not able and 10 representing fully able.

At the end of the quiz, users have another opportunity to immediately connect via live online chat or text with the care navigation team, schedule an appointment via an integrated online scheduling app, or leave their contact information with preferred method of outreach (email, phone, or text).

### Care Navigation: Engagement, Assessment, and Referral

Once an individual’s contact information is submitted via the quiz, chat, or the appointment scheduler, participants are then considered to be an “active inquiry.” Staff are available generally between 9 am and 5 pm during weekdays to interact instantly with users online. They are also available intermittently on evenings and weekends and are expected to respond to users who leave contact information within 24 h. The appointment scheduling process is designed to advance help-seeking by encouraging users who leave contact information to participate in a remote clinical assessment. The team initially reaches out via the user’s preferred contact method to thank them for their interest and to ask when they might be available to discuss their stated concerns. If users do not respond within 24 h, we continue to reach out systematically for the first 4 weeks, followed by enrolling users in our email drip campaign in an effort to maintain a connection. Once assessed, if clinically indicated, users are then offered a referral to local mental health resources based on their needs, location, and preferences. Referrals are enabled throughout NYS by a comprehensive third-party database of available mental healthcare and social service providers.

### Informed Consent and Safety Protocols

Verbal informed consent is obtained from individuals who interact with program staff via phone, text, email, live chat, or video call. Participants who provide consent are then provided with a description of the study. Study objectives are also described in detail in the privacy policy available on the campaign website. Ongoing data security is managed by complying with industry standards including The Health Insurance Portability and Accountability Act (HIPAA) and best practices at Northwell Health, where NYWell is hosted on a secure server. Participants who interact with research staff are assessed for safety. Incoming messages from participants are also monitored by research staff daily during standard business hours, and at least once daily during weekends. Any identified safety risk is immediately reported to the clinicians on the research team including a child and adolescent psychiatrists (MB) and licensed mental health counselor (NG) and escalated as necessary, including attempts to promptly connect the participant to appropriate local services.

### Data Collection and Analysis

Demographic and clinical data entered by each participant completing the quiz are collected. In addition, data is collected regarding the timing, frequency, and method (text, email, phone, live chat, video call) of each contact between the care navigation team and participants interacting with the campaign. Campaign analytics are also utilized to record digital ad and website engagement data including the amount of time spent on the website, the number of clicks while on the website, as well as user behavior while on the website such as completing the quiz, appointment scheduling, and live chat. Based on user responses to the online quiz, we identified three diagnostic risk categories (risk for psychosis, risk for depression, and risk for anxiety). In our analysis we selected a threshold such that reporting any of the adapted screener items pertaining to one of the three diagnostic categories suggested a risk state for that condition.

As part of the assessment process, we collect location data (city or neighborhood) whenever possible, enabling identification of local resources, and providing a measure of the socio-economic status of those referred. The American Community Survey income data available through the Economic Research Service and the City of New York was used to understand the complexion of those referred at the city and neighborhood level (32, 33). Based on these sources, low income was defined as neighborhoods or cities with a poverty rate of 20% or higher, or median family income less than 80% of median family income for the state or metropolitan area.

We used percentages, medians, and interquartile range (IQR) for descriptive statistics. Given symptom overlap between diagnostic risk categories (i.e., most participants report symptoms or experiences pertinent to more than one condition), we performed logistic regressions to analyze the relationship between each risk category (depression, anxiety, psychosis) and dichotomous variables (i.e., leaving contact information, reporting a desire to obtain a referral to care) while holding all other risk categories constant. Since we could not assume a normal distribution of contact response time, Mann-Whitney U test was performed to examine the difference in contact response time between those referred to services and those who were not.

## Results

### User Journey From Ad Impression to Inquiry

**FIGURE 3:**
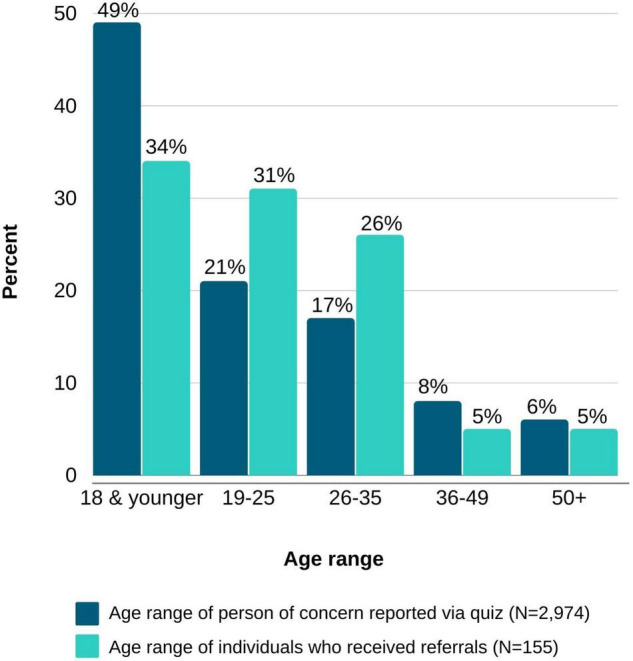
Age range reported via quiz (*n* = 2,974) compared to the age range of individuals referred to care (*n* = 155).

The user journey from digital ad (impression) to active inquiry is presented in Table 2. Between October 22, 2020, and July 31, 2021, the campaign resulted in a total of 581,981 ad impressions, 16,665 (2.9%) ad clicks, 13,717 (2.4%) unique website visitors, and 793 (0.1%) active inquiries. Altogether, the click through rate (percentages of clicks resulting from total number of impressions) of Google ads was approximately triple the rate of social media generated clicks. Instagram outperformed Facebook ads, representing 78.1% of social media-based clicks. In total, 69.3% (403,039/581,981) of impressions targeted youth searching for information on behalf of themselves, who eventually comprised 64.1% (8,798/13,717) of the total campaign website visitors. According to campaign analytics (available from Google for only 48.4% of website visitors), 71% of Youth and 69% of Allies accessed the site via desktop, while 27% of Youth and 31% of Allies accessed via mobile device. Youth were more engaged with the platform, spending on average 158 s on the website compared to 107 s for Ally visitors. Further, 45.5% of Youth campaign website visitors completed the quiz compared to only 11.4% of Ally visitors, and 7.6% of youth left contact information for the care navigation team compared to 2.4% of Ally visitors. Among quiz takers, nearly half (49%, 1,443) were 18 and younger, 21% (610) were 19-25, 17% (517) were 26-35, 8% (232) were 36-49, and 6% (172) were 50 and older (Figure 3).

**TABLE 2:** User journey from digital ad to active inquiry.

Online funnel	Total		YOUTH		ALLY	
			
	N	%^a^	N	%^a^	N	%^a^
**Impressions**						
Google Ads impressions	136,771	24%	77,052	19%	59,719	33%
Social impressions	445,210	76%	325,987	81%	119,223	67%
Total Impressions	581,981	100%	403,039	100%	178,942	100%
**Ad clicks**						
Google Ad clicks	7,877	5.8%^b^	4,712	6.1%^b^	3,165	5.3%^b^
Social Ad clicks	8,788	2.0%^c^	6,865	2.1%^c^	1,863	1.6%^c^
Total Clicks	16,665	2.9%	11,577	2.9%	5,028	2.8%
**Website activity**						
Unique Users	13,717	2.4%	8,798	2.2%	4,919	2.8%
Average time on page (seconds)	124	N/A	158	N/A	107	N/A
Quiz takers	4,562	33.2%^d^	4,002	45.5%^d^	560	11.4%^d^
Inquiries	793	5.7%^d^	673	7.6%^d^	120	2.4%^d^

*^a^ When not stated otherwise, the percentage is calculated out of the total number of impressions for the relevant column; ^b^ Percentage out of the Google Ad impressions; ^c^ Percentage out of the social-media impressions; ^d^ Percentage out of the number of unique users for the respective column.*

### Quiz Completers; Responses and Desired Outcomes

**FIGURE 4:**
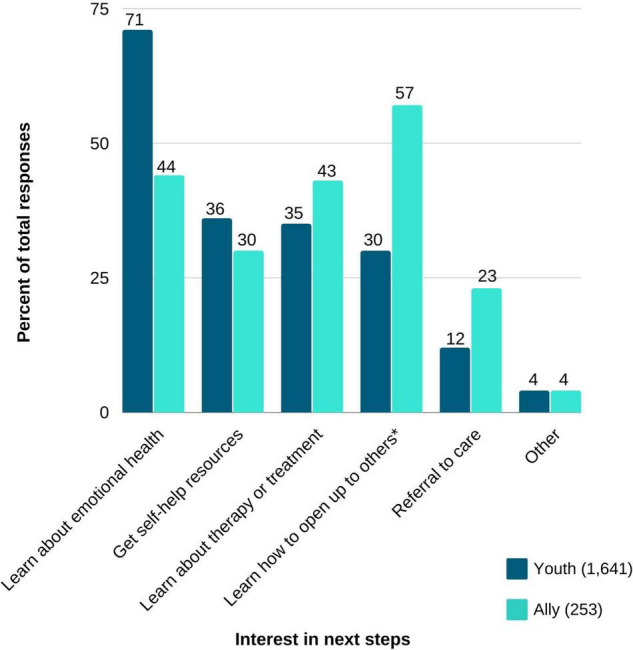
User preferences grouped by youth and allies (*n* = 1,894). *Ally selection was worded, “Learn how to talk to or support a loved one.”

Approximately one third of website visitors (4,562, 33.2%) completed the quiz. Responses, grouped by diagnostic risk categories are presented in Table 3. Most participants reported symptoms consistent with a risk for depression (93% youth and 84% ally). Psychosis risk was the least commonly reported diagnostic category (68% Youth and 60% Ally). Altogether (Figure 4), the most commonly reported desired outcome was to learn about emotional health (71% of youth and 44% of allies) and the least commonly reported goal was to receive a referral to care (12% youth and 15% ally). On average, users rated their ability to engage in work, school, relationships to be 5.35 (SD = 2.31) on a scale from 1 (unable) to 10 (fully capable). Approximately 16% of quiz-completers left contact information. Beyond the quiz, users most frequently visited the FAQ section of the website (1,050, 7.6%).

**TABLE 3:** User preferences grouped by diagnostic risk categories.

		Symptoms reported	Left contact information for follow up	Completed “next steps” questions (next columns)	Learn about emotional health	Learn how to open up to others; how to ask for help *^a^*	Learn about therapy or treatment	Obtain self-help resources	Referral to care
									
		N (%)	N (%)	N (%)	N (%)	N (%)	N (%)	N (%)	N (%)
Youth	**Total Youth Quizzes**	**4002 (100%)**	**624 (16%)**	**1641 (41%)**	**1171 (71%)**	**584 (36%)**	**569 (35%)**	**584 (36%)**	**199 (12%)**
	No psychosis symptoms	1284 (32%)	185 (14%)	474 (37%)	316 (67%)	115 (24%)	181 (38%)	176 (37%)	76 (16%)
	One or more psychosis symptoms	2718 (68%)	439 (16%)	1167 (43%)	855 (73%)	382 (33%)	388 (33%)	408 (35%)	123 (11%)
	No depression symptoms	296 (7%)	22 (7%)	88 (30%)	64 (73%)	12 (14%)	28 (32%)	25 (28%)	6 (7%)
	One or two depression symptoms	3706 (93%)	602 (16%)	1553 (42%)	1107 (71%)	485 (31%)	541 (35%)	551 (35%)	193 (12%)
	No anxiety symptom	758 (19%)	77 (10%)	283 (37%)	203 (72%)	72 (25%)	87 (31%)	104 (37%)	31 (11%)
	Anxiety symptom	3244 (81%)	547 (17%)	1358 (42%)	968 (71%)	425 (31%)	482 (35%)	480 (35%)	168 (12%)
Ally	**Total Ally Quizzes**	**560 (100%)**	**92 (16%)**	**253 (45%)**	**112 (44%)**	**143 (57%)**	**108 (43%)**	**76 (30%)**	**59 (23%)**
	No psychosis symptoms	226 (40%)	34 (15%)	97 (43%)	46 (47%)	54 (56%)	46 (47%)	31 (32%)	25 (26%)
	One or more psychosis symptoms	334 (60%)	58 (17%)	156 (47%)	66 (42%)	89 (57%)	62 (40%)	45 (29%)	34 (22%)
	No depression symptoms	126 (23%)	13 (10%)	58 (46%)	35 (60%)	24 (41%)	21 (36%)	14 (24%)	11 (19%)
	One or two depression symptoms	473 (84%)	79 (17%)	195 (41%)	77 (39%)	119 (61%)	87 (45%)	62 (32%)	48 (25%)
	No anxiety symptom	161 (29%)	19 (12%)	62 (39%)	25 (40%)	37 (60%)	32 (52%)	20 (32%)	11 (18%)
	Anxiety symptom	399 (71%)	73 (18%)	191 (48%)	87 (46%)	106 (55%)	76 (40%)	56 (29%)	48 (25%)

*^a^In the ally quiz, the wording of this question was altered to reflect a caregiver perspective, “Learn how to talk to or support a loved one.”*

Logistic regressions were performed to explore associations between diagnostic risk categories and the likelihood of leaving contact information and reporting a desire to receive a referral to care. Regressions were performed separately for both Youth and Ally campaign groups. While holding all other predictor variables constant, Youth visitors reporting symptoms consistent with risk for depression were twice as likely to leave contact information compared to those who did not report symptoms of depression (OR = 2.18, 95% CI [1.39, 3.41]); Youth visitors reporting symptoms consistent with a risk for anxiety were 69% more likely to leave contact information compared to those who did not report symptoms of anxiety (OR = 1.69, 95% CI [1.31, 2.19]). Though not statistically significant, the percentage of Youth visitors leaving contact information was lower in those who reported symptoms consistent with psychosis risk (11%) compared with those who did not report risk for psychosis (16%). Subsequently, reporting symptoms suggestive of psychosis among Youth was associated with a significant decrease of more than 40% in the likelihood of stating an interest in a referral to care (OR = 0.58, 95% CI [0.43, 0.80]). Endorsing depression or anxiety risk did not impact the likelihood of stating an interest in a referral to care.

Among Ally visitors, no significant relationship was found between diagnostic risk categories and the predictor variables, while holding other variables constant. However, a similar pattern to the one seen among Youth visitors emerged among Ally visitors (Table 3). For example, Ally visitors reporting symptoms of psychosis were descriptively less likely to state an interest in a referral to care compared to those not reporting psychosis risk (22% vs. 26%, respectively); Ally visitors endorsing risk for either depression or anxiety had descriptively higher interest in receiving a referral to care (25% vs. 19% and 25% vs. 18%, respectively).

### Care Navigation From Inquiries to Referrals

The trajectory from inquiry (leaving contact information) to assessment and referral, grouped by initial inquiry channel and participant (Youth vs. Ally) is presented in Table 4. Of the 793 inquiries that left contact information, 173 (21.8%) were successfully engaged to complete a virtual assessment. Most assessments resulted in a referral to care (155/173; 89.6%), indicating that those who successfully connected with the care navigation team were found to be experiencing psychiatric symptoms that warranted a referral.

**TABLE 4:** Trajectory from inquiry to assessment and referral, grouped by channel and participant.

	Total		Quiz		Video		Appointment scheduling		Pop up		Phone call		Ask us anything		Text messaging	
								
	N	%	N	%	N	%	N	%	N	%	N	%	N	%	N	%
**Total sample**																
Inquiries	793	100%	719	100%	23	100%	18	100%	14	100%	4	100%	14	100%	1	100%
Assessments	173	21.8%	139	19.3%	9	39.1%	11	61.1%	5	35.7%	3	75.0%	5	35.7%	1	100.0%
Referrals	155	19.5%	123	17.1%	9	39.1%	11	61.1%	4	28.6%	3	75.0%	5	35.7%	0	0.0%
**Youth**																
Inquiries	673	100%	633	100%	15	100%	14	100%	7	100%	0	0.0%	5	100%	0	0.0%
Assessments	130	19.3%	112	17.7%	7	50.0%	7	50.0%	2	28.6%	0	0.0%	2	40.0%	0	0.0%
Referrals	117	17.4%	100	15.8%	7	50.0%	7	50.0%	1	14.3%	0	0.0%	2	40.0%	0	0.0%
**Ally**																
Inquiries	120	100%	86	100%	8	100%	4	100%	7	100%	4	100%	9	100%	1	100%
Assessments	43	35.8%	27	31.4%	2	25.0%	4	100.0%	3	42.9%	3	75.0%	3	33.3%	1	100.0%
Referrals	38	31.7%	23	26.7%	2	25.0%	4	100.0%	3	57.1%	3	75.0%	3	33.3%	0	0.0%

Overall, the percentage of inquiries that eventually resulted in an assessment and referral was almost twice as high among Ally visitors (31.7%) compared to Youth visitors (17.4%). Of note, however, the number of visitors entering the Youth campaign was substantially larger compared to the Ally campaign, and thus most referrals to care were made via the Youth campaign. Most referrals (93.5%, 145/155) were to local general outpatient mental health services, while a minority were directed to first episode psychosis programs (3.9%, 6/155) and to clinical high-risk for psychosis programs (3.2%, 5/155). The median age of those referred was 21 years (IQR = 11). Compared to the age ranges reported in the quiz (*n* = 2,974), the referred population (*n* = 155) included a higher proportion of individuals 19–25 and 25–35, and a lower proportion of individuals 18 and under, 36–49, and 50 and older (Figure 3). Location data (city or neighborhood) was available for 79% (122/155) of the participants referred to care. Of those, 40% (49/122) were located in areas defined as low income.

### Contact Characteristics of Those Who Received a Referral to Care

As seen in Table 4, inquiries resulting in a referral to care was highest among users who initially made contact using phone call (*n* = 3/4, 75%) or appointment scheduling (*n* = 11/18, 61.1%), with the lowest percentage found in users submitting first inquiries via the quiz (*n* = 155/793, 19.5%). Of those who were referred to care, the median number of outreach attempts initiated by the care navigation team was 6 (IQR = 4). The median time difference from first inquiry to last contact prior to receiving a referral was 14.9 days (IQR = 22.3). The team’s initial response time after receiving an inquiry was significantly shorter for those who were successfully referred to care (median = 0.2 h, IQR = 9.8) compared to those who were not referred to care (median = 12.3 h, IQR = 28.7; Mann–Whitney *U* = 17,403, *P* < 0.001).

## Discussion

This manuscript aimed to characterize online help-seeking behaviors of youth and their allies interacting with a digital care navigation platform in NYS. Our findings suggest that self-reported symptomatology impact trajectories to care, even at the earliest stages of help-seeking, while youth and their allies are searching for information online. An online care navigation team could serve as an important resource for individuals with emerging behavioral health concerns and help to guide and support the transition between online information seeking at baseline to care.

Prior efforts to accelerate treatment initiation (34–38) have involved broad-based strategies to screen for mental illness within the community and/or raise awareness of the benefits of early intervention. Though educational campaigns have demonstrated success, they have relied predominantly on offline mass media channels, such newspaper advertisements, transit advertising, brochures, posters, TV, movie, and radio commercials. Websites were developed, though primarily designed for healthcare professionals and/or as a source of obtaining information about the project, rather than generating community-based referrals. Mindmap, a more recent federally funded research initiative in the United States (37), included digital media channels such as Facebook, Twitter, YouTube, Reddit, and LinkedIn, in addition to traditional mass media, though this strategy was also primarily intended to contribute to education rather than generate referrals directly. Mindmap inquiries were largely made by phone rather than interactive online channels, and referral sources were predominately clinical rather than self-generated community referrals. Our findings are in line with growing literature highlighting the potential for digital technology to improve the process of identifying struggling youth and the effectiveness of outreach and engagement efforts (22, 24, 39–41). The Internet may serve as a critical resource to connect with concerned youth and their allies at the earliest possible time in the help-seeking trajectory, when individuals first begin to search for information online. While many individuals describe their first contact with psychiatry as a negative and often traumatic experience (42), online resources, staffed by peers and mental health professionals, such as the NYWell project offers an opportunity to ease the introduction to care, paving the way for greater engagement with services and thus improved outcomes for youth with behavioral health conditions.

In line with prior reports (19–22, 43), we found that younger participants were predominantly interested in obtaining behavioral health information rather than a referral to care. Their quiz responses and online behaviors reflect a desire to understand their experiences and obtain answers to questions and concerns. For example, while completing our symptom quiz was a popular online activity among youth, once completed, most youth did not leave contact information, demonstrating limited readiness to take action toward receiving support. Furthermore, while youth ages 18 and under represent nearly half of all quiz completers, most referrals to care were provided to young adults between the ages of 19–25 and 26–35. This discrepancy may be partially explained by the requirement for minors to obtain parental consent to engage in psychiatric care in NYS, representing another potential barrier to help seeking. Importantly, our data highlight that self-reported symptomatology impacts the likelihood of advancing help-seeking behaviors beyond information gathering. This pattern was apparent for both youth and their allies. Understanding the barriers and facilitators present to each diagnostic group and more granularly to each individual, will be critical to improving efforts to reduce the duration of untreated illness. Tailored strategies will likely be necessary to advance help seeking of those in need based on a thorough appreciation of their symptoms and obstacles to accessing care. For example, while substantial stigma is associated with psychosis, depression and anxiety are somewhat less stigmatized (44, 45) and may partially explain the differences in the likelihood of reporting a desire to seek care and to leave contact information. It is also plausible that symptoms associated with depression and anxiety are more readily identifiable, as exemplified by evidence supporting online assessment of these conditions (46, 47), while symptoms of psychosis are more challenging to recognize, contributing to delayed help-seeking (48). Lastly, the presence of common psychotic symptoms themselves, such as paranoia, may result in mistrust of our efforts and rapid disengagement.

Youth were highly interested in completing our self-assessment quiz, however, most did not leave contact information and were lost to follow up, highlighting both a strength and limitation to online resources. While the Internet provides a comfortable and anonymous setting for help-seeking (49), our ability to confidently ascertain who these individuals are and understand their motivation for searching online and completing a self-assessment quiz is limited. Further, we are unable to clinically confirm the presence or absence of self-reported psychiatric symptoms based on the quiz alone. While psychiatric self-screeners effectively assess for psychiatric diagnoses risk (29–31), few self-screeners have been validated in an online environment (50, 51), which may consist of a different population and setting. In order to build better online resources for youth and their allies, we will likely need strategies informed by symptomatology, and further validating online self-screeners delivered over the Internet will be a critical next step.

Our findings highlight that individuals online present at very different stages along the help-seeking continuum (52) and in varying degrees of readiness for change at the time of online search query. While some were immediately prepared to take action by proceeding with a remote clinical assessment and referral, many others were reluctant to advance and either did not leave contact information or were lost to follow up. Further, motivation for help-seeking likely oscillates over time. For instance, we found that a quicker response time was more likely to result in a referral, perhaps capturing a critical window of opportunity when help-seeking motivation was higher. Future research will need to build agile online interventions and creative engagement strategies designed to meaningfully advance help-seeking behaviors based on an individual’s needs and readiness for action at the moment of initial engagement. A successful care navigation platform will ultimately need to ascertain, beyond presenting symptoms, which individuals might benefit most from what kind of online support and guidance based on where they are at that moment in the help-seeking trajectory. Further, separate strategies will need to be developed and tested for allies. Our data support the critical importance of engaging allies in facilitating treatment initiation as a greater proportion were ready to take action compared to youth. Further, while youth in our dataset readily interacted with our digital ads and gravitated toward completing an online self-assessment, allies were much less likely to engage in either of these activities, preferring instead to expeditiously connect to a licensed professional. More research is necessary to understand how to effectively reach and engage allies online.

Forty percent of our referred population with known neighborhood or city data were in low-income areas. By comparison, New York State’s poverty rate is 12.7%, and New York City’s is 17.9% suggesting that our platform may be reaching and serving a disproportionate number of individuals with limited financial means (32, 33). Collecting a range of additional demographic data in future implementations of the platform will enable us to ascertain the potential for a youth-focused online care navigation service as a mechanism to improve access to care for populations who are underserved due to social, economic, policy, and environmental factors (53).

### Limitations and Future Directions

There are several noteworthy limitations. First, we selected to develop and implement a self-assessment quiz, designed to function as an engagement tool. We did not utilize validated symptom screeners, limiting our ability to report with greater certainty on the mental health characteristics of the population we reached. Further, given substantial symptom overlap between the symptoms reported in each of the three diagnostic risk categories, future research will need to better delineate how specific diagnoses and psychiatric symptoms impact online help-seeking behaviors for both youth and allies. This may require the development of novel digital symptom self-screeners deployed in an online environment, or the incorporation of established self-screeners, followed by a remote clinical assessment to confirm diagnostic accuracy or determine the most effective threshold for accurately identifying symptoms in an online environment. Second, most participants opted to remain anonymous, and many others were lost to follow up, limiting our ability to determine what these individuals want, need, and how best to support them. Although, we can reasonably assume that these individuals are interested in obtaining mental health related information, we are unable to confirm their motivation, if they are in need a referral to traditional psychiatric care, or other forms of sub-clinical support. Many website visitors likely do not need formal psychiatric intervention and future research will need to explore novel engagement and intervention strategies for those who might benefit from support and guidance and whose symptoms do not meet the threshold necessary to warrant formal intervention from a mental health professional. Additionally, the success of online treatment programs aimed at young people (54–56) may offer innovative and effective alternatives to traditional in person care. Fourth, the NYWell project was designed ultimately to identify individuals in the earliest stage of help-seeking and expeditiously refer them to care. This approach was successful at capturing a momentary spike in help-seeking motivation, while individuals are searching online for information, however, the campaign demonstrated limited ability to maintain help-seeking motivation over time for many in order to advance them from online information seeking at baseline to community-based care. Accordingly, this approach may have resulted in individuals being lost to follow up, who require alternate forms of engagement, or a longer timeline over which to gain the awareness, self-efficacy, and social support necessary to take action. Much more work is required between these two endpoints (identification and referral) and tailored engagement efforts will be critical to meaningfully advance help-seeking. Future interventions will need to incorporate flexible and personalized strategies based on an individual’s readiness for change, needs, and expectations while incorporating lessons learned thus far such as the importance of response times and selecting the most appropriate digital platforms for each target audience. Lastly, given that the project is still active at the time of publication, data related to cost were not included. Campaign initiation is typically more expensive until algorithm optimization occurs on the digital advertising platform, which is a dynamic and ongoing process. Once the campaign is complete, a formal cost effectiveness analysis will be conducted to accurately present associated costs.

Leveraging digital technology, an online care navigation platform may prove to be a critical resource capable of refining the help-seeking process for youth and their allies. Our findings reinforce the need to further delineate how individuals progress beyond online information seeking at baseline to meaningfully taking action toward care. By better understanding motivations and barriers, we can continue to expand and implement tailored engagement strategies, designed to effectively support and guide youth and their allies with mental health questions and concerns. Moreover, we must continue to develop and test digital support tools and interventions geared toward the growing population of youth who are in need of support, but not yet ready, and may not need, formal psychiatric care delivered by a mental health clinician.

## Data Availability Statement

The original contributions presented in the study are included in the article/Supplementary Material, further inquiries can be directed to the corresponding author.

## Ethics Statement

The studies involving human participants were reviewed and approved by Northwell Health IRB. Written informed consent from the participants’ legal guardian/next of kin was not required to participate in this study in accordance with the national legislation and the institutional requirements.

## Author Contributions

MB, CG, JK, AB, and LD conceptualized and executed the project and interpreted the results. NG, DS, CL, and KF contributed to participant recruitment and data collection. AB conducted the data analysis along with support from CG and MB. MB, AB, and CG wrote the initial draft of the manuscript. All authors contributed to the manuscript preparation and editing.

## Conflict of Interest

The authors declare that the research was conducted in the absence of any commercial or financial relationships that could be construed as a potential conflict of interest.

## Publisher’s Note

All claims expressed in this article are solely those of the authors and do not necessarily represent those of their affiliated organizations, or those of the publisher, the editors and the reviewers. Any product that may be evaluated in this article, or claim that may be made by its manufacturer, is not guaranteed or endorsed by the publisher.

## Supplementary Material

Supplementary Figure 1 Strong 365 NYWell Youth Website.
