# Supplementary figures and images for: Digital Strategies to Accelerate Help-Seeking in Youth With Psychiatric Concerns in New York State
Source: Front Psychiatry. 2022 May 16;13:889602. doi: 10.3389/fpsyt.2022.889602 (PMC9157179; doi:10.3389/fpsyt.2022.889602)

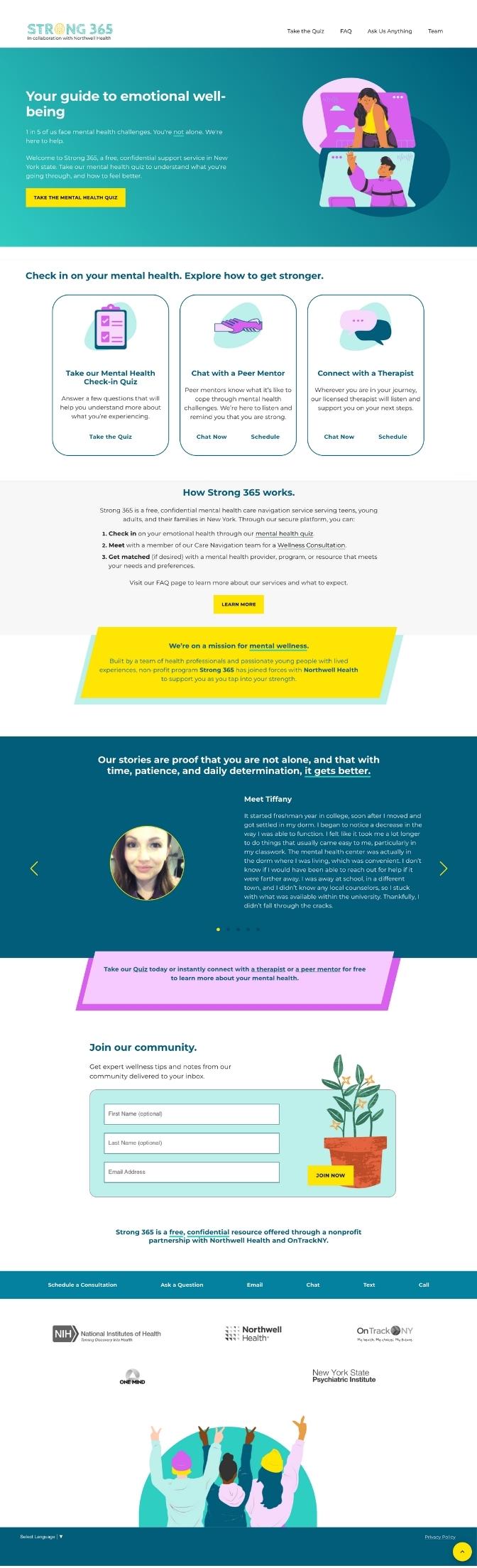

Supplement: Supplementary Figure 1 — Strong 365 NYWell Youth Website. [file Image_1.JPEG]
